# Experiences of Individual Care Workers in Oral Care of Dementia Patients

**DOI:** 10.3390/medicina60071087

**Published:** 2024-07-02

**Authors:** Evelina Daugėlienė, Karolina Skučaitė, Jurgita Andruškienė, Šarūnė Barsevičienė, Eglė Žymantienė

**Affiliations:** Faculty of Health Sciences, Department of Dental Hygiene, Higher Education Institution Klaipedos Valstybine Kolegija, LT-92255 Klaipeda, Lithuania; skucaite.k@gmail.com (K.S.); jurgita.andruskiene@gmail.com (J.A.); s.barseviciene@kvk.lt (Š.B.); e.zymantiene@kvk.lt (E.Ž.)

**Keywords:** dementia, oral cavity care, experiences, individual care, elderly population

## Abstract

*Objective*: Dementia is one of the most common diseases in the elderly population. The aim of this study was to reveal the experiences of individual care workers performing oral care for dementia patients. The oral health of the elderly is in a critical state and in most cases not enough attention is paid to this problem. *Material and methods*: A qualitative research method was chosen for the research. The data collection instrument was a semi-structured interview, the purpose of which was to reveal the experience, knowledge, and attitude of individual care workers towards the importance of oral care and the oral hygiene habits of patients with dementia. The number of research informants was 10. The inclusion criteria were individual care workers working in social care institutions who were responsible for the personal care of dementia patients. *Results*: The analysis of the study showed that the main obstacles facing individual care workers in performing oral care for dementia patients are related to their cognitive disorders. For this reason, unreasonable fears of patients may arise, which also affect the proper performance of individual oral hygiene and other tasks assigned to employees. *Conclusions*: The results of the study showed that according the informants’ opinions it is crucial to take care of dementia patients’ oral care daily.

## 1. Introduction

Dementia is one of the most common diseases in the elderly population. According to the WHO, the number of people diagnosed with this disease is constantly increasing. It is estimated to reach 75 million by 2030, and a large proportion of cases of this disease are undiagnosed [1,2]. According to the Hygiene Institute, in 2023, 41,689 residents with dementia were registered in Lithuania [3].

Dementia is a result of a variety of diseases and injuries that affect the brain. Alzheimer’s disease is the most common form of dementia and may contribute to 60–70% of cases [1]. Dementia is a progressive neurodegenerative disease characterized by the impairment of cognitive functions such as spatial perception, memory and thinking, as well as difficulties in performing routine tasks in daily activities. The aging population and the emergence of co-morbidities, such as dementia, prompt action to ensure the well-being of this population. One of the main goals of preventive dentistry is to preserve the healthy teeth of elderly patients [4].

The oral health of the elderly is in a critical state and, in most cases, there is a lack of attention to this area. Elderly people and people with special needs often suffer from oral diseases. The most common problems, such as poor chewing and speaking functions, xerostomia and bad breath, can affect self-esteem and communication. Elderly people with dementia often present with a greater burden of oral diseases, with the prevalence of the latter more so evident in the advanced stages of the condition. Evidence demonstrates a higher incidence of dental caries, periodontal disease, increased pain and other related dental problems in dementia. People with mental disorders lose their independence in everyday life. It is important for such patients to constantly monitor their oral health status, so the help of other people, caregivers or health care professionals is needed to timely notice changes in oral health status, to ensure the proper personal oral hygiene care of patients with dementia, but many studies have shown that individual care workers fail to understand the importance of oral health [5,6]. Educational programs for individual care workers should be created and implemented by dental hygienists for the purposes of preventive oral care [5].

Adapting communication to the needs of individuals with reduced cognitive ability, as well as involving familiar caregivers, can prove challenging in everyday dental practice [7].

There is a lack of research on the experiences of individual care workers in the work environment. More attention is paid to the role of nurses and the burden they experience at work. However, nursing assistants are an integral part of the health care system, as they supervise and care for patients, as well as devote a lot of effort to those who cannot or can only partially care for themselves. Individual care workers are also responsible for the oral care of dementia patients. However, their knowledge, attitude on this issue and the obstacles they face in the oral care of patients with neurodegenerative and other serious diseases have not been extensively studied abroad, and no such studies have been conducted in Lithuania so far.

The aim of this study was to reveal the experiences of individual care workers performing oral care for dementia patients. 

## 2. Materials and Methods

A qualitative research method, applying semi-structured interviews, was used to collect research data. This method was expected to reveal, as accurately as possible, the experiences, knowledge, and attitudes of individual care workers towards the importance of oral cavity care and oral hygiene habits of patients with dementia. The questionnaire was created based on a scientific study conducted by Weening-Verbree et al. [8]. The content analysis was conducted to analyze the research data.

This study was conducted in June–October 2023. A total of 10 individual care workers (all of them female) who provide personal care to patients with dementia (in social care institutions) agreed to participate in this study (Table 1).

Prior to conducting this study, permissions were obtained from the administrations of two social care institutions, X and Y. According to the principle of justice, all informants participated in the survey voluntarily. Interviews were conducted respecting informed consent and confidentiality principles. All audio recordings were available only to the researchers. Individual interviews lasted for 15 min on average, were recorded and later transcribed.

The interview consisted of 12 questions: 1 introductory question, 9 in-depth questions, 1 closing question and 1 question to find out contextual, socio-demographic data. The questions can be divided into four parts according to the topics:

− The first part aims to reveal the experience of individual care workers in performing oral cavity care for patients with dementia (questions 1–4);
− The second part aims to assess the oral hygiene habits of dementia patients (questions 5–7);
− The third part aims to determine the knowledge and attitude of individual care workers towards the importance of care of the oral cavity of patients with dementia (questions 8–11);
− The fourth part aims to find out the socio-demographic indicators of the study participants—age, gender, work experience in the field (years), and education in obtaining the qualification of an individual care worker (question 12).

## 3. Results

### 3.1. Experiences of Individual Care Workers in Oral Cavity Care for Dementia Patients

At the beginning of the conversation, informants were asked “How do you feel today?” Three subcategories are distinguished according to the answers given and supporting statements (Table 2).

Most informants assessed their well-being positively (6). It is emphasized that this work requires feeling good so that employees can perform their tasks properly, and this is confirmed by the answer of the informant (M1): “I feel very good because we have to feel good during our work”. Other informants confirmed negative or ambivalent opinions (4). One person said that “well-being is good, but at the same time fatigue is also felt, because the work is really hard”. 

The aim of this study was to find out what challenges individual care workers face in oral care for dementia patients. In the analysis of the data from this study, five main supporting statements were identified (Table 3).

Individual care workers were most often faced with the reluctance of patients (6) to perform oral care. Individual care workers said that dementia patients do not accept help and some of them are hostile. They do not understand why they need to open their mouth or what is required of them. Physically, they could do it themselves, but due to their illness, it is difficult to do it. Four supporting statements were made in the subcategory that the performance of oral care is aggravated by patients’ fears. Residents protect their removable dentures, and they may hide them from staff, because they think that they will take them away. In addition, patients’ fear of water was mentioned. Two individual care workers said that the behaviors depend on the mood swings of the patients (2). This is reflected in the answer of the informant (M8): “one day the mood is good, and the next day he refuses to clean up”.

We wanted to know how the study participants dealt with problems that arose during oral care. The answer options provided by the individual care workers are grouped into five subcategories and supporting statements (Table 4).

Most informants indicated that they were dealing with problems related to the maintenance of the oral care in patients with dementia by trying to adapt to the timings of their better moods (4). This is confirmed by the answer of the informant (M5): “We try to approach patients when their mood is better.” Another way of solving the problem is persuading (3). Since individual care workers are faced with the problem that some patients do not understand how to brush their teeth and how to rinse, two informants used the method of interpretation (2). When a patient does not agree to accept help in oral care, the help of other persons is used (1) or dentures are picked up for cleaning when a patient is eating (1).

In our study, there was interest in how often patients with dementia can independently take care of their oral health. When analyzing the data of the study, patients’ abilities were divided into three subcategories with supporting statements (Table 5).

The informants report that most dementia patients already have an advanced stage of the disease when administered to the social care institution and are fully dependent on help (4); a smaller number of patients partially require help or are self-sufficient (4). This is confirmed by the answer of the informant (M1): “<…> perhaps 10 percent of the dementia patients would be able to do everything by themselves, when the disease is not far advanced <…>”.

### 3.2. Oral Hygiene Habits of Dementia Patients

Informants were asked to describe oral hygiene habits for dementia patients. According to the given answers, two categories were distinguished. The first describes oral hygiene habits and the second one discusses the tools used. We see the answers of the informants with the supporting statements in Table 6.

It was revealed that all individual care workers perform dental cleaning twice a day (10) “In the morning and in the evening”. Individual care workers were asking patients who wear removable dentures to remove them for cleaning (6). Eight informants reported mouthwashes being used in patients’ daily routines. This is confirmed by the answer of the informant (M8) “Who needs, who wants a relative brings mouthwashes”. And individuals who do not have mouthwash are usually offered to rinse or brush with tap water. This is supported by the informant’s (M7) statement: “I suggest rinsing with water <…>”. Patients’ behavior often complicates the process of dental cleaning causing a lack of interdental care (2). This is reflected in the informant’s (M1) response: “We don’t use the dental floss for those who have teeth because they refuse to use it”.

One of the main tools for dental care is toothpaste (10). Several examples of them were also cited as: “Colgate”, “Blend a med”, “Sensodyne”, and “Ecodenta”. Some individual care workers describe them as “<…> standard <…>”, “<…> the simplest and cheapest <…>”, and a few considered good pastes. However, one informant (M6) gave the answer that when there are patients in the social care institution who also use more expensive toothpastes, they have anti-inflammatory, anti-bleeding and other good active ingredients. The supporting statement for this is as follows: “One elderly woman with dementia is very much cared for by her son, he takes her to dentists and dental hygienists, brings her better toothpastes, like Parodontax”. Unfortunately, this is observed only in patients whose relatives are engaged more in their care. The results showed that toothpastes and toothbrushes were the tools with which dentures were cleaned (3). Four informants say that soft and hard toothbrushes are used. We noticed that the tools for oral care are provided by relatives of patients who often don’t pay attention to the quality/type of the toothbrush. The supporting statement of the subject (M1) is as follows: “<…> tools are bought by relatives, and they buy it just to be new and do not pay attention, if it is a soft or hard toothbrush. But when we ask to buy it, then discuss which one is better”. However, we see that there is an opportunity to ask relatives who are responsible for their family members to buy a soft brush, especially when there are bleeding gums. Although the subjects indicated that soft and hard toothbrushes are used in their institution, their opinions are that the toothbrush should be soft, because, as they note, such patients have gum problems. There have also been a couple of informants who reported that they usually use or at least try to use a soft toothbrush. One nurse’s assistant also gave examples of mouthwashes, like Eludril and Listerine. Because there are a lot of elderly people living in social care institutions and the majority wear removable dentures, patients with dementia may not be able to say that they have problems with it. This results in wounds and aches that employees are trying to heal. The informant (M5) explains how such a problem is solved: “If the removable dentures cause wounds, we wait until it heals and use the ointments <…>”. There are also particularly difficult cases when it is very difficult for a patient to carry out brushing their teeth, but cleaning is still necessary, so individual care workers resort to non-routine cleaning measures that are not even adapted for cleaning the dentures or mouth. Supporting this is the informant’s (M6) statement: “<…> with gauze and toothpicks because you’re still trying somehow to get out of the situation”.

This part discusses the use of additional tools such as denture glue and dissolving tablets for cleaning removable dentures. Considering the statements, three subcategories were formed. The informants’ answers to these questions are given in Table 7.

Most informants said they use denture glue (9). And as we can see from the answers given, it is often emphasized that not everyone uses them, because not everyone has them. The supporting statement of the informant (M1) is as follows: “For some patients, we use Corega denture glue if they have it”. We also note that Corega glue of the same type of removable dentures is usually used. Half of the individual care workers indicated that they were using these tablets. And the others claimed the opposite about their use, as indicated by the statement of the informant (M8): “We do not soak with tablets”.

The next question was to find out if care is taken to ensure patients’ dental examinations and professional dental hygiene. According to the statements, two subcategories were formed. We see the responses of the informants with supporting statements in Table 8.

The data of this study revealed that patients with dementia are rarely taken to oral care specialists preventatively (2). Most often, such patients visit these specialists when acute problems arise (3); this is confirmed by the answer of the informant (M8): “We take patients to dentists, dental hygienists, when the problem appeared, or hurts”.

### 3.3. Knowledge and Attitude of Individual Care Workers about the Oral Care of Dementia Patients

We asked if individual care workers had interacted with a dental hygienist by gaining knowledge about the peculiarities of oral care for dementia patients. If the answer was no, then they were asked to comment on who provided this knowledge. Based on the provided options, two categories were formed. The first category characterizes the experience of individual care workers in communicating with the dental hygienist, from which two subcategories are distinguished. The second category is comprised of other sources of information that provided knowledge about the care of the oral cavity of patients, divided into three subcategories with its supporting statements (Table 9).

The results showed that while some individual care workers claimed to be visiting oral care professionals, six informants reported that they were not inclined to consult on this issue. One of this study’s informants (M1) gave the statement: “I visit the dental hygienist, but I have not been able to communicate on this issue”. Four informants were consulted by dental hygienist on the matter. The informants are willing to expand their knowledge by attending seminars and using their own or colleagues’ experiences (6). As one of the informants (M1) states, “We have certain seminars held, not every year, but our institution sends us to improve ourselves in certain courses. It happens that we learn something in the courses”. Three informants obtained qualifications needed for oral care in professional school (3). According to one of the study participants (M4), “During my professional training I was taught about oral care of people with dementia<…>”.

During the interview, individual care workers were asked if they had enough knowledge of the peculiarities of oral care for dementia patients, as well as whether they would like to receive additional training from a dental hygienist in order to gain more knowledge. According to the data provided by the individual care workers, two categories were formed. The first category describes the opinion of individual care workers about the amount of their knowledge related to the peculiarities of oral care for dementia patients. The next category discusses the need of individual care workers to receive additional training from a dental hygienist to gain more knowledge. Both categories have two subcategories each, with supporting statements (Table 10).

Four informants claimed to have enough knowledge, but six expressed the need to be additionally trained about the peculiarities of oral care for dementia patients. One informant’s (M3) response confirms as follows: “I wouldn’t want to, there’s enough knowledge. Because we use what relatives bring. We won’t go and buy those tools ourselves”. Another informant’s (M9) supporting statement was as follows: “It would be good to have more of them. Especially about the care of dentures”.

All individual care workers understand the importance of oral health (M1–M10). According to their responses, two categories have been formed. The first presents the opinions of informants about the importance of daily care of the patient’s oral health. The second category describes how individual care workers understand the need for daily oral care for patients with dementia (Table 11).

The informants gave many reasons as to why oral care was necessary. Nine informants emphasized the importance of daily oral care for dementia patients. Most often in this subcategory it was noted that the goal is to avoid bad breath. The second most common reason was due to the comfort of the patient and their proper quality of life (7). The main focus was to preserve the remaining teeth of patients and their ability to chew. The supporting statement for this is as follows: “<…> because when patients arrive, we see, that the teeth wasn’t brushed at all, then these people have a lot of problems, and we have to fix them, they have brought it to us and we have to do everything: to do new dentures, to heal the teeth”. One informant’s (M5) statement reveals that there is a sense of duty, a responsibility for each patient to take care of their oral cavity, and at the same time an understanding of what patients feel and how much it affects their quality of life. Several informants claimed that it is important for prevention and the reduction of health problems (3). These arguments were associated with a reduction in the risk of developing inflammatory processes.

At the end of the interview, the informants were asked what factors could improve the quality of oral care for dementia patients. When analyzing the data from this study, the listed factors of informants were divided into four subcategories with supporting statements (Table 12). 

Five informants suggested that appropriate tools for dental hygiene should be provided. The second suggestion was to reduce the workload (4). Additional organization of training (3) for individual care workers was also a relevant topic. We have already discussed the situation about training (Table 9). However, a few informants also mentioned that it would certainly be useful if more training could be organized. And the other three informants in this study stated that nothing needs to be changed. 

Answering the main question of this study about the experience of individual care workers with dementia patients in oral care, it can be concluded that the main obstacles that arise are related to patients’ behavioral and cognitive disorders that interfere with the performance of oral care. Even more challenging, such individuals no longer understand why they need to open their mouths or what is required of them, and unreasonable fears and mood swings appear. It was revealed that informants are under too much workload pressure. All these factors interfere with the proper performance of individual oral hygiene and other tasks. When discussing the knowledge and attitude of individual care workers to the importance of oral care for dementia patients, it was found that the workers quite correctly understand and approve of the importance of performing daily oral care.

## 4. Discussion

The majority of the informants said that they felt good when they were asked about their experiences while taking care of dementia patients (6). According to Eisenmann et al. [9], taking care of people with dementia is becoming more and more challenging with time, not only for the family members but also for the health care workers. This was also confirmed by several informants of this study (3), who said they felt good, but at the same time they felt tired because their work was difficult.

The main obstacles while performing oral care procedures for patients with dementia, as stated by the individual care workers, were analyzed. The obstacle most frequently named by the informants was the unwillingness (6) of patients to perform oral care. The other obstacles were the lack of patients’ thinking and reduced skills (4). The patients needed much explanation about how to rinse or reminding how to place the toothbrush in the mouth. These statements are corroborated by the findings of other scientists [8,10], which have revealed that a major part of being an individual care worker is having a challenging experience while working with patients with cognitive impairment.

The results of our study are similar to the findings of other researchers [8], which have concluded that individual care workers have heavy workloads (2).

According to Van Manen et al. [11], the only way for nursing staff to meet the needs of persons with cognitive impairment is efficient communication and well-developed nonverbal communication skills.

Our study revealed that the greatest factor in patients’ inability to take care of themselves independently was the advanced stage of dementia. It has also been confirmed by other scientists that the ability to take care of themselves is dependent on the stage of disease.

It was confirmed by Gao et al. [12] that efficient dental hygiene was one of the most important means to prevent the development of periodontal diseases. The results of our study revealed that individual care workers were brushing patients’ teeth twice per day (10), “In the morning and in the evening”.

The findings of our study, showing that patients were experiencing difficulties while using an interdental brush, were in line with Gao et al.’s [13] study, which concluded that patients with dementia frequently experience difficulties while performing individual dental hygiene, due to the symptoms of dementia.

The results of our study showed that the most frequent reason why dementia patients were taken to the dentist was related with urgent needs for treatment, not prevention. This could be explained and confirmed by the findings of Geddis-Regan et al. [14], saying that systemic diseases progress alongside dementia, and it also worsens oral health status.

According to Weening-Verbree et al. [8], in most cases individual care workers are taking care of dementia patients’ oral care; however, in most cases, the dental hygiene status of the patients with dementia is insufficient.

The majority of informants (7) said that they had enough knowledge; however, some of the informants (3) said they could have more information about oral health care. It was also confirmed by Manchery et al. [6] that the efficient education of nursing staff about the relation between oral care and development of oral diseases could improve the attitude of nursing staff towards oral care importance.

The results of our study were in line with the study of Weening-Verbree [8], which revealed that the staff of nursing homes understood the importance of oral care.

Options for improving the quality of the oral health care of dementia patients was analyzed in our study, and it was revealed, and also confirmed by other researchers, that supply of the proper tools for oral care (5) is important, as well as a reduced workload (4) for those who are taking care of the dementia patients.

### Study Limitations

1. Small sample size: one of the primary limitations of this qualitative study is the small sample size of ten participants. While qualitative research focuses on in-depth exploration and understanding of individual experiences, the generalizability of findings may be limited due to the small number of participants.
2. Selection bias: the participants selected may not be fully representative of the target population, leading to potential biases in the data collected and the themes identified.
3. Limited scope and depth: with only ten participants, the depth and breadth of data collected in the study may be limited. Certain nuances, variations, or subcategories within the data set may not be fully explored or adequately represented.
4. Potential for response bias: with a small sample size, there is a risk of response bias, where participants may be more inclined to provide socially desirable responses or may not fully disclose their experiences or perspectives.

Future research could explore similar research questions with larger sample sizes to enhance the robustness and applicability of the findings.

## 5. Conclusions

The results of this study showed that, in the informants’ opinions, it is crucial to address the daily needs of dementia patients’ oral health. The main obstacles of individual care workers in performing oral care for dementia patients are related to their cognitive disorders. For this reason, unreasonable fears of patients may arise, which will also affect the proper performance of individual oral hygiene and other tasks assigned to employees. According to the opinion of the individual care workers, most of them claimed that the knowledge they have gained is enough, but they would not mind receiving additional training from an oral hygienist in order to gain more information.

## Figures and Tables

**Table 1:** Informant age, work experience and identification codes.

Nr.	Age	Years of Experience	Identification Code
1.	57 y	13 y	M1
2.	58 y	15 y	M2
3.	57 y	3 y	M3
4.	59 y	14 y	M4
5.	59 y	14 y	M5
6.	58 y	14 y	M6
7.	45 y	5 y	M7
8.	62 y	30 y	M8
9.	55 y	38 y	M9
10.	52 y	6 y	M10

**Table 2:** The well-being of individual care workers.

Category	Subcategory	Supporting Statements
Assessment of the well-being of individual care workers (10)	Positively evaluated (6)	“Excellent.” (M2) “Amazingly.” (M7) “Working mood.” (M8) “Okay.” (M9), (M10) “I feel very good because we have to feel good during work.” (M1)
	Negatively evaluated or ambivalent (4)	“Tired.” (M3) “Very tired.” (M4) “Anyway.” (M6) “Okay, but I’m very tired, feeling fatigue.” (M5)

**Table 3:** Challenges experienced by individual care workers in oral care for dementia patients.

Category	Subcategory	Supporting Statements
Challenges faced by individual care workers in oral care for dementia patients (18)	Patient mood swings (2)	“Others, when they don’t have the mood, don’t listen.” (M5) “With mood swings, one day the mood is good, and the next day refuses to clean their teeth.” (M8)
	Reluctance of patients (6)	“<…> they don’t want to give and don’t want to take out their prostheses.” (M1) “Not everyone gives them, doesn’t open their mouths. <…> Gives the dentures only to a relative. (M3) “It happens that it does not allow to remove dentures, can even bite. Or when I touch their mouth, they start yelling that they’re being beaten. When I perform oral care, they don’t want to put on dentures, they don’t want to hold them when they’re taken out, they push them out with their tongues themselves.” (M5) “<…> don’t let to do the mouth examination.” (M7) “When they don’t want, we don’t do.” (M9) “It happens that they refuse to brush their teeth and are angry.” (M10)
	Loss of cognitive skills (4)	“There’s one resident who doesn’t understand the need for oral care. That’s why it takes a lot of time to persuade to take out the dentures.” (M2) “Not everyone knows how to rinse and spit out after brushing their teeth” (M4) “They can’t all rinse, they swallow water. We say to rinse, but they don’t understand.” (M5) “Most of the time, people don’t realize they need to rinse with water. Sometimes they brush their teeth holding the toothbrush with the other end. You have to stand and show everything you can do.” (M6)
	Patient fears (4)	“It seems to them that this is their thing, their teeth, so they protect their dentures very strongly.” (M1) “Patients are hiding, not giving away dentures because they think that we are going to steal them.” (M2) “Some residents wrap up their dentures in paper because they think somebody want to steal it. Hide in places where it’s really hard to find.” (M5) “Others are afraid of the water.” (M7)
	High workload (2)	“High workload.” (M7) “A little bit overload.” (M8)

**Table 4:** Ways to solve problems that have arisen during oral care.

Category	Subcategory	Supporting Statements
Ways to solve problems that have arisen during the peculiarities of oral care (11)	Method of persuasion (3)	“Most of the time, we do it through dialogue. Through dialogue, you try to convince a person that this is necessary, and we have to do it <…> We’re talking, we’re cleaning up.” (M1) “We persuade.” (M2) “You talk to a person; you motivate as much as you can.” (M8)
	Method of interpretation (2)	“It has to be explained for those who can clean themselves that they need to rinse and spit because others swallow.” (M4) “We stand with them… we talk…, show how to do it.” (M6)
	Targeting a better mood of patients (4)	“We try to reach out to patients when their mood is better.” (M5) “Accessible according to their moods.” (M7) “After a while, we try again when the mood is better.” (M3) “After a while, we try again.” (M9)
	Other methods, e.g., when eating or using help of others (2)	“When eating, we take it and clean it”. (M7) “We invite the person on duty, and he helps us. Of course, we are trying as hard as we can.” (M10)

**Table 5:** The ability of patients with dementia to take care of their oral health independently.

Category	Subcategory	Supporting Statements
The ability of patients with dementia to take care of the oral health independently (8)	Self-sufficient or needs partial assistance (4)	“<…> perhaps 10 percent of the dementia patients would be able to do everything by themselves, when the disease is not far advanced <…>” (M1) “We still have to remind the person, to help.” (M1) “<…> you have to say you need to rinse the mouth; you need to spit the water out.” (M4) “It all depends on the stage of the disease; you need to stand and encourage.” (M5)
	Fully dependent on help (4)	“We’re basically taking care of it.” (M2) “It depends on the stage of the disease, how advanced it is, when for patient is difficult to clean, we do it, <…> Most don’t clean themselves.” (M4) “Practically never. Most can’t take care of themselves.” (M6) “There are no ones who can take care of themselves.” (M7)

**Table 6:** Features of oral hygiene habits in patients with dementia and the used tools.

Category	Subcategory	Supporting Statements
Oral hygiene habits (26)	Brushing twice a day (10)	“<…> if there are teeth of our own, we perform in the morning and evening.” (M1) “In the morning and in the evening.” (M2), (M4), (M5), (M6), (M7), (M8), (M9), (M10). “In the morning and in the evening we perform toothbrushing.” (M3)
	Cleaning of the removable dentures (6)	“When a person has dentures, we ask them to take them out and we wash it <…>.” (M1) “In the morning, we put the dentures clean, and in the evening we clean them again.” (M2) “Whoever has dentures please take them out, we clean and put them in.”(M5) “Whoever has removable dentures, we clean it <…>.” (M6) <…> we clean the dentures of course.” (M7) “We clean dentures <…>.” (M10)
	Rinse with mouthwashes (8)	“<…> mouthwash.” (M3) “<…> mouthwash.” (M4) “<…> we’re rinsing too.” (M5) “Whoever has inflammation, rinse <…>. Mouthwashes “Listerine” and “Eludril”.” (M6) “I suggest rinsing with water <…>.” (M7) “Whoever needs it, who wants relatives brings mouthwashes to those who want it.” (M8) “Some rinse with mouthwashes.” (M9) There are very few patients who have mouthwashes, unless they’re able to take care of themselves.” (M10)
	Do not use dental floss (2)	“We don’t use the dental floss for those with teeth because they’re not going to let us to use it.” (M1) “We don’t use the dental floss.” (M2)
Tools used in the care of the oral health (21)	Soft toothbrush (2)	“We try to use the soft brush because those who have teeth should maintain them clean.” (M4) “The brushes are mostly soft because we ask relatives to buy soft brushes.” (M6)
	Toothbrush soft and hard (4)	“<…> the toothbrush is bought by relatives, and they buy it just to be new and do not pay attention is it soft or hard.” (M1) “The toothbrush is bought by relatives, so the brush is hard or soft. <…> if it’s too hard, then we ask relatives to buy another one.” (M5) “Brushes are all sorts.” (M8) “Toothbrushes are what we get.” (M10)
	Toothpaste (10)	“Toothpastes Colgate, Blend a med, I don’t remember further.” (M1) “We use toothpaste <…>.” (M2) “Toothpaste <…>.” (M3), (M4) “Every resident has a toothpaste <…>. Toothpaste “Colgate”, “Sensodyne”.” (M5) “We use toothpastes that relatives bring. Most often the simplest and the cheapest. One elderly woman with dementia is very much taken care of her son, who takes her to dentists and dental hygienists, she has better toothpastes, like Parodontax.” (M6) “<…> a few teeth left is brushed with toothpaste <…>.” (M7) “Toothpastes are good to us, like Blend a med.” (M8) “Toothpaste “Colgate”.” (M9) “We have good toothpastes as Ecodenta, Colgate.” (M10)
	Cleaning of the removable dentures with toothpaste and toothbrush (3)	“With the toothpaste and toothbrush, we clean the dentures of course.” (M7) “Wash with toothpaste <…>.” (M8) “We clean dentures with a brush and toothpaste.” (M10)
	Other measures (2)	“If the dentures rubbed the gums, we wait for it to heal, apply ointments <…>.” (M5) “<…> we clean with gauze and toothpicks because we have somehow to get out of the situation.” (M6)

**Table 7:** The use of additional tools for removable dentures in dementia patients.

Category	Subcategory	Supporting Statements
Additional tools are used for removable dentures (19)	Denture glue is used (9)	“For some residents, we use Corega denture glue if they have one.” (M1) “We use glue, but there’s little who has it.” (M2) “We’re sticking to those who are still conscious.”(M3) “We use Corega glue.” (M5) “We use glue, <…>” (M6), (M7) “Some use glue.” (M8) “Some stick with glue.” (M9) “We’re sticking with Corega who have it.” (M10)
	Dissolving tablets are used (5)	“Only one other has dissolving tablets to clean dentures, which ones we have and use. Otherwise, we wash it with running water and leave it to dry.” (M1) “Yes, dissolve the tablets and soak in the liquid.” (M2) “We also use soluble tablets, who has it, who needs it." (M5) “<…> and dissolve the tablet once a week.” (M6) “<…> we use tablets if they are bought by the relatives, but more for lying patients.” (M7)
	Dissolving tablets are not used (5)	“You don’t really have <…>.” (M3) “Soake in the water <…>.” (M4) “We don’t soak with tablets.” (M8) “We don’t use dissolving tablets.” (M9) “We don’t use dissolving tablets <…>.” (M10)

**Table 8:** Visiting oral care professionals in dementia patients.

Category	Subcategory	Supporting Statements
Visits to dental care specialists (5)	Taken to oral care specialists when problems appear (3)	“We take patients with dementia to dentists if necessary.” (M2) “When something’s more serious, we take them to the dentists. If it hurts, we call to the doctor.” (M7) “We take patients to dentists, dental hygienists, when the problem is already there, or it hurts.” (M8)
	Taken to oral care specialists for prevention purposes (2)	“One patient has periodontitis, we can’t touch her, she starts yelling, so we let her clean her teeth herself. Sometimes we take her to the dental hygienist, or the family members take her.” (M5) “One elderly women’s with dementia son takes care of her and takes her to the dentists and dental hygienists when needed <…>.” (M6)

**Table 9:** The experiences of individual care workers in communicating with dental hygienists and gaining knowledge about the peculiarities of oral care for patients with dementia.

Category	Subcategory	Supporting Statements
Dental hygienist as a source of information that has provided knowledge about dementia patients’ oral care (10)	Communicated with the dental hygienist (4)	“I’ve got the knowledge from the dental hygienist and a dentist.” (M2) “Yes.” (M4) “I do my dental hygiene and I get some knowledge from my dental hygienist.” (M5) “I had to this procedure.” (M10)
	Didn’t communicate with the dental hygienist (6)	“I visit the dental hygienist, but I haven’t been able to communicate on this issue.” (M1) “I didn’t <…>.” ((M3), (M8), (M9) “No.” (M6), (M7)
Other sources of information that have provided knowledge about dementia patients’ oral care (14)	From seminars and courses (5)	“We have certain seminars, not every year, but our institution sends us to develop into certain courses. It happens that we learn something in the courses.” (M1) “I’m improving through courses.” (M2) “I learned more about patient oral care from courses that we go to very often.” (M5) “We are sent to the courses to improve and learn.” (M6) “From the course <…>.” (M7)
	At the time of obtaining the profession (3)	“When I got a profession, I was taught about oral care when people have dementia <…>.” (M4) “When I was learning that getting this qualification taught us.” (M6) “I was taught in the professional school about it.” (M8)
	On their own experience (6)	“I’m discussing about dental care with family members.” (M1) “I gained knowledge through experience from colleagues.” (M3) “From practice the most.” (M3) “<…> from experience.” (M7) “I learned everything from experience.” (M8) “From experience.” (M9)

**Table 10:** The knowledge of individual care workers and the need to gain more information about the peculiarities of oral care for dementia patients.

Category	Subcategory	Supporting Statements
Opinion of individual care workers on the amount of their knowledge related to the peculiarities of oral care for dementia patients (10)	Sufficient knowledge (7)	“I personally have enough of that knowledge.” (M1) “I think I have enough knowledge.” (M2) “It seems that’s enough.” (M3) “I have enough.” ((M5), (M7). “Enough.” ((M6), (M10).
	Insufficient knowledge (3)	“There could be more knowledge.” (M8) “Knowledge is never too much.” (M4) “It would be good to have more of them.” (M9)
The need of individual care workers to receive additional training from a dental hygienist to gain more knowledge (10)	Needs more training (6)	“I’d like to be trained, certainly not against it.” (M1) “Knowledge is never too much, everything improves.” (M4) “Of course I would.” (M7) “I wish there were more trainings.” (M8) “It would be good to have more of them. Especially about the care of dentures.” (M9). “There could be training.” (M10)
	Does not need more training (4)	“<…> don’t need any more, I know everything, what more could be invented.” (M2) “Maybe that training would be good, but I don’t think “I’d learn something new.” The way we work, dental hygienists don’t work that way <…>” (M6) “I wouldn’t want, there’s enough knowledge. Because we use what relatives bring. We won’t go and buy those tools ourselves.” (M3) “I wouldn’t want to; we go to courses very often.” (M5)

**Table 11:** Attitudes and awareness of individual care workers about the importance of performing oral care for dementia patients.

Category	Subcategory	Supporting Statements
Awareness of individual care workers about the reasons why daily oral care is important for dementia patients (19)	For general condition of the body and for hygiene (9)	“<…> the odour from the mouth, the chewing, depends on it.” (M1) “<…>You need to rinse because of the bacteria in your mouth.” (M2) “For the bacteria to disinfect.” (M3) “It’s good for health because it’s all connected.” (M4) “<…> and smell bad.” (M5) “The mouth is clean, there is no bad smell, to be, for the sake of health.” (M6) “Bacteria accumulate.” (M7) “Still for hygiene, for the smell.” (M8) “<…there’s no smell <…>.” (M9)
	Prevention and health problems reducing (3)	“<…> so that there are no inflammations.” (M1) “<…> to avoid tooth loss.” (M2) “You need to take care, if not later toothache could appear <…>.” (M5)
	For the comfort of the patient and the proper quality of their life (7)	“I would like a person to keep their remaining teeth for as long as possible. It is better for the person himself when he can eat normally, <…>.” (M1) “Also, to be able to chew and make the well-being better.” (M2) “You need to brush the remaining teeth.” (M3) “<…> because when patient arrive, we see, that the teeth haven’t been brushed at all, then these people have a lot of problem, and we have to fix them.” (M5) “<…> want patients to be with their teeth.” (M6) “To keep the teeth as long as possible, because the other teeth are healed, so we have to keep them in the mouth.” (M8) “<…> and it’s better for the person himself when his mouth is clean.” (M9)

**Table 12:** Factors that could improve the quality of oral care for dementia patients based on the opinion of individual care workers.

Category	Subcategory	Supporting Statements
Opinion of individual care workers on factors that could improve the quality of oral care for dementia patients (15)	Reduced workload (4)	“To slow down the workload <…>.” (M1) “Less workload.” (M4) “To slow down the workload.” (M7) “<…> and that workload could be less.” (M8)
	Additional training (3)	“More training about dentures care.” (M6) “<…> to do more training.” (M7) “Could do more training for us, <…>.” (M9)
	Appropriate tools for dental hygiene (5)	“There could be more and better tools for cleaning.” (M1) “Provide the right tools.” (M4) “So that everyone has the necessary tools.” (M6) “For everyone, soft toothbrushes wouldn’t mix.” (M7) “Tablets should be used to clean dentures.” (M9)
	Nothing needs to be changed (3)	“I’m happy with everything.” (M2) “I’m not missing anything <…>.” (M3) “I wouldn’t change anything.” (M5)

## Data Availability

The data presented in this study are available on request from the corresponding author. The data are not publicly available due to ethical concerns.
